# Supraglottic jet oxygenation and ventilation assisted fiberoptic intubation in a paralyzed patient with morbid obesity and obstructive sleep apnea: a case report

**DOI:** 10.1186/s12871-019-0709-7

**Published:** 2019-03-20

**Authors:** Hansheng Liang, Yuantao Hou, Huafeng Wei, Yi Feng

**Affiliations:** 1 Department of Anesthesiology, Peking University People’ s Hospital, Beijing, 100044 China; 2 Department of Anesthesiology and Critical Care, Hospital of the University of Pennsylvania, Philadelphia, PA 19104 USA

**Keywords:** Supraglottic, Jet ventilation, Oxygenation, Obesity, OSA, Fiberoptic bronchoscope, Intubation, Difficult airway

## Abstract

**Background:**

Hypoxia is a major concern and cause of morbidity or mortality during tracheal intubation after anesthesia induction in a pathological obese patient with obstructive sleep apnea (OSA). We introduce a case using Supraglottic jet oxygenation and ventilation (SJOV) to promote oxygenation/ventilation during fiberoptic intubation in a paralyzed patient with morbid obesity and OSA.

**Case presentation:**

A 46-year-old man weighting 176 kg with BMI 53.7 kg/m2 was scheduled for gastric volume reduction surgery to reduce body weight under general anesthesia. SpO2 decreased during induction, and two hand pressured mask ventilation partial failed. We then placed WEI Nasal Jet Tube (WNJ) in the patient’s right nostril to provide SJOV. Then fiberoptic bronchoscopy guided endotracheal intubation was performed via mouth approach, and vital signs were stable. The operation was successfully completed after 3 h. Patient recovered smoothly in hospital for 8 days and did not have any recall inside the operating room.

**Conclusion:**

SJOV via WNJ could effectively maintain adequate oxygenation/ventilation during long time fiberoptic intubation in an apnea patient with morbid obesity and OSA after partial failure of two hand pressured mask ventilation, without obvious complications. This may provide a new effective approach for difficult airway management in these patients.

## Background

The pathological difficult airway usually place patients in danger during general anesthesia induction [1], which may result in a high percentage of airway-related morbidity and mortality [2, 3]. Hypoxia is a major concern and cause of morbidity or mortality during tracheal intubation after anesthesia induction in a pathological obese patient with obstructive sleep apnea (OSA) [4–6]. A new approach to promote oxygenation/ventilation has been described during various difficult airway managements (*Peng J,Xie P,Wu CN,Wei HF,*et al.) [1, 7–11] and under propofol anesthesia (*Su DS,BJA 2017*), especially in obese patient. We introduce a case using Supraglottic jet oxygenation and ventilation (SJOV) to promote oxygenation/ventilation during fiberoptic intubation in a paralyzed patient with morbid obesity and OSA.

## Case presentation

A 46-year-old man weighting 176 kg with BMI 53.7 kg/m2 was scheduled for gastric volume reduction surgery to reduce body weight under general anesthesia. The patient was diagnosed of obstructive sleep apnea (OSA) 3 years ago, without treatment. Airway inspection showed short neck with circumference of 51 cm, limited neck extension due to its thick fat and the Mallampatti score-Ш. The patient felt tired preoperatively because of his sleep deprivation secondary to OSA. He was very nervous and refused to consent for awake fiberoptic intubation under sedation.

We elected to perform tracheal intubation after anesthesia induction but keeping patient’s spontaneous breathing to avoid hypoxia, with initial direct laryngoscopy using video laryngoscope, and back up with fiberoptic intubation and then laryngeal mask airway (LMA). Bispectral index (BIS) was used to monitor anesthesia depth.

**Fig. 1:**
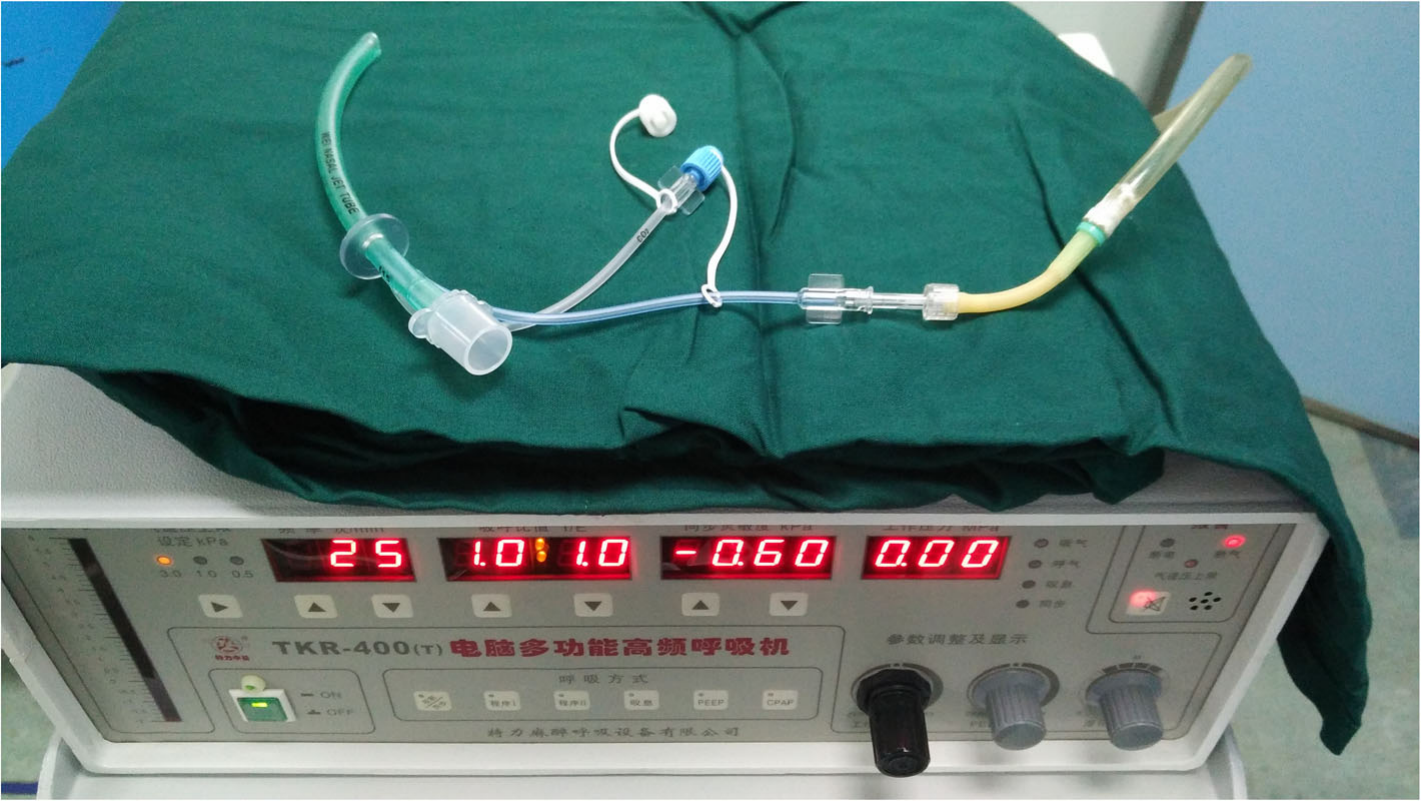
Components and assembly of the WEI Nasal Jet Tube (WNJ) and automatical jet ventilator: P_et_CO_2_ = end tidal CO_2_ pressure (Compliance with ethical standards: The automatical jet ventilator-TKR-400 and WNJT upon examination and approval by the company, pictures can be used)

Vital signs showed Bp 142/79 mmHg, HR 88 bpm, SpO2 96%, RR 22 bpm before anesthesia induction. Midazolam 3 mg and sufentanil 10 μg was given intravenously to reach BIS at 62 for sedation. Thereafter, intravenous 100 mg propofol was given and BIS fell to 51. Mask pressurized ventilation could be performed to maintain SpO2 100% with patient under continuous target controlled infusion (TCI) at propofol 3μg/mL.Direct laryngoscope with video laryngoscope was tried twice but failed because of the invisible glottis obstructed by Huge epiglottis (Grace IIb view). SpO2 fell to 75% at the end of second laryngoscopy. Two hand pressurized mask ventilation was initiated and became difficult, although SpO2 could be maintained around 88% with following vital signs: BP 133/73 mmHg, HR 86 bpm, normal sinus rhythm, BIS 57.We then placed WEI Nasal Jet Tube (WNJ), (Well Lead Medical Equipment Ltd., Guangzhou, China. Production batch number: 20140901) (Fig. 1) in the patient’s right nostril to provide SJOV. The jet catheter of the WNJ was connected to an automatical jet ventilator-TKR-400 (Well Lead Medical Equipment Ltd.,Guangzhou, China.) with following working parameters: driving pressure (DP) 35 psi, respiratory rate (RR): 55 bpm, I/E ratio 1:3.SpO2 began to rise again and reached 100% at 1 min after initiation of SJOV. Thoracic cage moved ups and downs during SJOV indication of both oxygenation and ventilation. We then administered intravenous rocuranium 60 mg, TCI propofol 4μg/mL and controlled ventilation was achieved using SJOV. Fiberoptic bronchoscopy guided endotracheal intubation was performed via mouth approach. Fiberoptic intubation was difficult due to hypertrophy of the patient’s tongue and epiglottis but eventually succeded 5 min later. The vital signs at the end of successful intubation were as followings: SpO2 100%,BP 125/64 mmHg, HR 71 bpm, sinus rhythm, BIS 45.PetCO2 was not monitored during fiberoptic intubation due to the both Ports being not consistent and hurry to raise oxygenation. However,instant blood gas analysis showed pH 7.36, PaO2 124 mmHg and PaCO2 49 mmHg. Total time of SJOV via WNJ was about 7 min. No obvious barotrauma, nose bleeding etc.,was seen at the end of intubation.

The operation was successfully completed after 3 h. The patient was transferred to the ICU and was extubated in ICU without event. Patient recovered smoothly in hospital for 8 days and did not have any recall inside the operating room.

## Discussion and conclusion

This case clearly demonstrated the SJOV via WNJ could effectively maintain adequate oxygenation/ventilation during long time fiberoptic intubation in an apnea patient with morbid obesity and OSA after partial failure of two hand pressured mask ventilation, without obvious complications. This may provide a new effective approach for difficult airway management in these patients.

According to the world health organization (WHO) standards established 1989, body mass index (BMI) ≧28 is defined as obesity, BMI≧40 is defined as morbid obesity [12]. Adipose tissue accumulated in pharyngeal can make patient’s pharyngeal cavity narrow and make the oropharyngeal muscles and soft tissue collapse, obstructing the airway [12, 13]. Obviously, this increases the patient’s workload of breathing and reducing functional residual capacity, causing ventilation/perfusion mismatch,eventually hypoxia and intolerance of hypoxia [14]. The incidence of difficult intubation in morbid obesity patients during general anesthesia induction undergoing abdominal surgery can be as high as 24% [15], and the awake intubation may be required in about 8% of patients [15]. The potential upper respiratory tract obstruction in OSA patient contribute to its high perioperative complication rate at about 19.6% [16]. The incidence of failed intubation in OSA patients is10 times higher than normal [17], while the incidence of intubation failure about patients with morbid obesity and OSA increases to 100 times [17, 18]. We discussed awake intubation with this patient on pre-anesthesia visit, but the patient was nervous and could not be comforted and refused the plan. In addition, the patient’s large neck circumference makes it difficult to identify the cricothyroid membrane. Since we underestimated the difficulty of this patient’s airway, and this patient was very nervous and unwilling to be stimulated, we did not choose awake intubation or awake look. Even though this case was an unanticipated difficult airway, we were able to maintain and improve oxygenation using WEI nasal jet tube for supraglottic jet oxygenation and ventilation,because we have some experiences applying this device on obese patients. We have completed a randomized controlled study(Registration number, ChiCTR 1,800,017,028) of supraglottic jet ventilation to improve oxygenation in obesity patients, and the results revealed that Nasal Jet tube could prevent patients suffering from desaturation during propofol-remifentanil protocol undergoing hysteroscopy. We are in the process of writing a paper.

The most important features of supraglottic airway is most characteristic of the patient with pathological obesity and OSA is serious airway collapse after anesthesia due to hypertrophy of the patient’s tongue,epiglottis and supraglottic muscles [19]. Airway collapse make supraglottic airway narrower, making the air flow of the mask pressurized ventilation difficult to pass the narrow area [20]. WNJ can pass the narrow airway and support the collapse and resolve the ventilation problem. WNJ is designed to be soft to void injury of nasal mucosa damage and bleeding. The evaluation of nasal cavity and the use of the lubricant also ease its insertion and minimizing nose bleeding. In our case, nasal jet tube maintained this patient’s oxygenation. According to the “Difficult Airway Society 2015 guidelines for management of unanticipated difficult intubation in adults” [21], muscle relaxant can make tracheal intubation easier, and prevent laryngospasm, so we administered rocuronium. Succinylcholine should be a better choice in this case, but we don’t have it in our department. With quick and short-acting effect, rocuronium could exert muscle relaxant effect 60 s after administration, which is equivalent with succinylcholine.

The capacity of tolerance to hypoxia in pathological obese patients with OSA is reduced significantly. It is critical in this case to have an effective method to maintain the patient’s oxygenation during intubation because of the shorter Oxygen Saturation Falling Time (OSFT) [22]. The definition of OSFT is the time of the patient’s SpO2 dropped to 90% after full oxygenation 100% oxygen inhaled and removed nitrogen [22, 23]. The obese patient’s OSFT is only 163 s due to the increased oxygen consumption and the changes in the airway described above,but the average of a normal adult is 526 s [22–24]. So it is almost impossible to complete this fiberoptic intubation without maintaining effective oxygenation using SJOV via WNJ for 7 min in this anesthetized and paralyzed morbid obese patient with OSA. This patient didn’t reach “can’t intubate and can’t ventilate”(CICV) so that LMA placement was not tried, nor did the emergent tracheotomy was considered.

In combination with previous publications [1, 7–11], It seems that SJOV has following advantages compared to the transtracheal jet ventilation (TTJV) recommended by American Society of Anesthesiologists (ASA) guidelines for the management of difficult airway. First,SJOV via WNJ is less invasive than TTJV which may increase the incidence of barotrauma barotrauma [7, 19]. Secondly, it usually take longer time to perform effective TTJV than placement of WNJ in the nose and could be very difficult to achieve successful TTJV in obese patient with thick neck. Thirdly, it is convenient to monitor ventilation using the built-in port for PetCO2 monitoring in WNJ. Forth and most importantly, SJOV maintain the open feature of airway during jet ventilation, minimizing or preventing the severe barotrauma, often seen during the use of TTJV, especially in emergent difficult airway management [1].

In summary, SJOV via WNJ seems to be an effective new approach to maintain adequate oxygenation/ventilation during difficult airway management in an apnea patient with morbid obesity and OSA.

## Abbreviations

ASA: American Society of Anesthesiologists
BIS: Bispectral index
BMI: Body mass index
CICV: Can’t intubate and can’t ventilate
DP: Driving pressure
LMA: Laryngeal mask airway
OSA: Obstructive sleep apnea
OSFT: Oxygen Saturation Falling Time
SJOV: Supraglottic jet oxygenation and ventilation
TCI: Target controlled infusion
TTJV: Transtracheal jet ventilation
WHO: World health organization
WNJ: WEI Nasal Jet Tube
